# Fixed Allele Differences Associated With the Centromere Reveal Chromosome Morphology and Rearrangements in a Reptile (*Varanus acanthurus* BOULENGER)

**DOI:** 10.1093/molbev/msad124

**Published:** 2023-06-05

**Authors:** Jason Dobry, Zexian Zhu, Qi Zhou, Erik Wapstra, Janine E Deakin, Tariq Ezaz

**Affiliations:** Institute for Applied Ecology, University of Canberra, Canberra, Australia; MOE Laboratory of Biosystems Homeostasis and Protection and Zhejiang Provincial Key Laboratory for Cancer Molecular Cell Biology, Life Sciences Institute, Zhejiang University, Hangzhou, China; MOE Laboratory of Biosystems Homeostasis and Protection and Zhejiang Provincial Key Laboratory for Cancer Molecular Cell Biology, Life Sciences Institute, Zhejiang University, Hangzhou, China; Center for Reproductive Medicine, The 2nd Affiliated Hospital, School of Medicine, Zhejiang University, Hangzhou, China; Evolutionary & Organismal Biology Research Center, School of Medicine, Zhejiang University, Hangzhou, China; School of Natural Sciences, University of Tasmania, Hobart, Australia; Institute for Applied Ecology, University of Canberra, Canberra, Australia; Institute for Applied Ecology, University of Canberra, Canberra, Australia

**Keywords:** chromosome rearrangements, fixed alleles, centromere, evolution, cytogenetics, chromosomics

## Abstract

Chromosome rearrangements are often implicated with genomic divergence and are proposed to be associated with species evolution. Rearrangements alter the genomic structure and interfere with homologous recombination by isolating a portion of the genome. Integration of multiplatform next-generation DNA sequencing technologies has enabled putative identification of chromosome rearrangements in many taxa; however, integrating these data sets with cytogenetics is still uncommon beyond model genetic organisms. Therefore, to achieve the ultimate goal for the genomic classification of eukaryotic organisms, physical chromosome mapping remains critical. The ridge-tailed goannas (*Varanus acanthurus* BOULENGER) are a group of dwarf monitor lizards comprised of several species found throughout northern Australia. These lizards exhibit extreme divergence at both the genic and chromosomal levels. The chromosome polymorphisms are widespread extending across much of their distribution, raising the question if these polymorphisms are homologous within the *V. acanthurus* complex. We used a combined genomic and cytogenetic approach to test for homology across divergent populations with morphologically similar chromosome rearrangements. We showed that more than one chromosome pair was involved with the widespread rearrangements. This finding provides evidence to support de novo chromosome rearrangements have occurred within populations. These chromosome rearrangements are characterized by fixed allele differences originating in the vicinity of the centromeric region. We then compared this region with several other assembled genomes of reptiles, chicken, and the platypus. We demonstrated that the synteny of genes in Reptilia remains conserved despite centromere repositioning across these taxa.

## Introduction

Genomic differentiation across the genome is often variable within species, with some regions displaying high levels of differentiation and other regions showing lower levels or no differentiation. The heterogeneous pattern of genomic divergence has been broadly observed across taxa (Harrison 1991; Orr and Turelli 2001; Wu 2001; Nosil et al. 2009; Ortiz-Barrientos et al. 2016) and has been termed “genomic islands of divergence” (Wu 2001; Nosil et al. 2009; Berg et al. 2017). Chromosomal rearrangements can drive heterogeneous divergence within the genome by suppressing recombination of the loci on the rearranged chromosome (Livingstone and Rieseberg 2004; Faria et al. 2019a, 2019b; Huang and Rieseberg 2020; Bedoya and Leaché 2021). When a chromosome rearrangement results in recombination suppression from misalignment, the result is a polymorphism of homologous chromosomes and heterokaryotypic individuals. One chromosome maintains the ancestral collinear state that continues to recombine within the population. The newly derived rearranged region, in contrast, has a sequence order void of allelic diversity, represented by a single haplotype for all the loci captured by the rearrangement (Wellenreuther and Bernatchez 2018; Faria et al. 2019a, 2019b). If the new arrangement has a selective advantage, it can spread to a frequency that allows the establishment of a parallel collinear state but with a derived sequence order (Westram et al. 2022). Within an established rearrangement, elevated rates of mutation occur relative to the ancestral collinear genome (Navarro and Barton 2003). For example, it was demonstrated that proteins on rearranged chromosomes evolved at twice the rate of the collinear genome between humans and chimpanzees (Navarro and Barton 2003; Rieseberg and Livingstone 2003). Chromosome polymorphism results in an ancestral chromosome block that maintains the collinear genome and a derived chromosome block that drives divergence (Faria et al. 2019a, 2019b). Maintenance of this polymorphism through balancing selection of haplotypes is likely a key mechanism for forming genomic islands of divergence (Nosil et al. 2009).

Understanding how genomes are structured and organized from genomic rearrangements has remained an important question since the beginning of the genome sequencing era (Eichler and Sankoff 2003). How do genomic islands form and how or why evolutionary breakpoints evolve through lineages whereas maintaining highly conserved motifs are not fully understood (Ingles and Deakin 2018; Deakin et al. 2019; Farré et al. 2019; Waters et al. 2021). Homologous syntenic blocks are arrangements of genes that maintain synteny across taxa and through evolutionary time. In the case of chromosome rearrangements, flanking regions are known as evolutionary breakpoint regions, which tend to be conserved across lineages (Murphy et al. 2005; Larkin et al. 2009; O’Connor et al. 2018; Farré et al. 2019; Damas et al. 2021, 2022). Syntenic blocks and their flanking evolutionary breakpoint regions are known for three significant features: unique regions unshared with other taxa, repeat elements, and conserved regions (Longo et al. 2009; Dobigny et al. 2017; Damas et al. 2021, 2022). Frequently reused repeat motifs characterize the breakpoint regions (Farré et al. 2015). Repeat regions populated with transposable elements, segmental duplications, and tandem repeats are fragile and dynamic regions that “live and die” within the genome (Alekseyev and Pevzner 2010; Farré et al. 2015). An essential aspect of understanding chromosome restructuring is that these regions tend to be located between genes, cluster at genome “hotspots,” and the reuse of these regions is nonrandom (Farré et al. 2016; O’Connor et al. 2018; Damas et al. 2019, 2021; Zhou et al. 2021).

Rearrangements alter chromosome structure by moving blocks of DNA sequence ranging from 130 kb to 100 Mb within the same chromosome or between chromosomes (Wellenreuther and Bernatchez 2018). These rearrangements can host thousands of genes and, as a result, have the potential to be evolutionary earthquakes (Peng et al. 2006; Farré et al. 2015). Rearrangements can form “supergenes” often associated with linked traits and coadapted alleles (Wellenreuther et al. 2014; Campagna 2016; Charlesworth 2016; Iijima et al. 2018; Adams and Castoe 2019; Merritt et al. 2020; Anderson and Tung 2021). They often occur in populations along ecotones or on the outer edge of the species distribution (Rieseberg 2001; Morales et al. 2019; Faria et al. 2019a, 2019b; Huang and Rieseberg 2020; Kess et al. 2021) and are also frequently associated with hybrid populations between closely related species (Marhold and Lihová 2006; Payseur and Rieseberg 2016; Bedoya and Leaché 2021; Potter et al. 2022; Dobry et al. 2023a, 2023b). Rearrangements are generally detected in four ways: 1) cytogenetically, if they capture structural features such as the centromere and alter the morphology of the chromosome, 2) through molecular cytogenetics, with fluorescent probes that hybridize to known positions on the chromosomes (inversions can be observed or putative rearrangements can be validated), 3) population genetic/genomic inferences such as linkage disequilibrium (Faria et al. 2019a, 2019b; Huang and Rieseberg 2020) and differences in population structure using PCA from patterns of allele frequencies that reflect the differentiation of specific loci (Li and Ralph 2019; Huang et al. 2020) ,and 4) bioinformatically using comparative genomics (Koochekian et al. 2022). However, bioinformatic and population genetic indicated regions are only putative unless verified cytogenetically (fluorescence in situ hybridization [FISH]).

Regions surrounding centromeres are known hotspots for rearrangement (Smalec et al. 2019; Ola et al. 2020; Nesta et al. 2021). The highly repetitive nature of the centromere makes it one of the most challenging regions within the genome for cloning and sequencing. The role of the centromere is critical for cell division, and significant disruptions to this process can lead to chromosome instability (Forsburg and Shen 2017) or result in fatal consequences (Lu and He 2019). Therefore, the characterization of the centromeric regions is of significant interest in biological studies and the presentation of genetic diseases (Holland and Cleveland 2012). One way to investigate the nature of centromere relocation involves the comparative analysis of individuals with centromere rearrangements. By identifying those loci in and around the region of interest and observing a pattern of characterizable genetic differences, we can add to the broader understanding of the roles these changes play in species adaptation (Yeaman 2013), evolution (Potter et al. 2022), and cancer (Alonso and Dow 2021).

The Varanidae are a group of reptiles occurring in Africa, the Middle East, Asia, the Indo-Pacific, and Australia. All species that have been karyotyped have 2*n* = 40 chromosomes with ZZ/ZW sex chromosomes (Dutt 1968; King and King 1975; De Smet 1981; King et al. 1982; Srikulnath et al. 2013; Matsubara et al. 2014; Pokorná et al. 2016; Patawang et al. 2017; Iannucci et al. 2019a, 2019b; Augstenová et al. 2021; Dobry et al. 2023a). Whereas chromosome number is highly conserved in this family, there is an interspecies variation of chromosome morphology, but generally, this variation classifies species groups from widespread geographical areas. For example, all Asian species have the same chromosomal morphology, but two karyotypes are found within Africa (King and King 1975; Iannucci et al. 2019a, 2019b). Australia has the most remarkable species diversity, with 34 species currently described (Uetz et al. 2021) and three different chromosomal morphotypes exist within Australia. These correspond to species clusters from three clades defined by these karyomorphs—the Gouldii clade, Varius clade, and Odatria clade which diverged rapidly within the last 15 Ma (fig. 6) (Brennan et al. 2021). Within Odatria are the ridge-tailed goannas represented by a species complex of taxonomic ambiguity spanning over 100 years (Boulenger 1885; Mertens 1942; Storr 1966, 1980; King et al. 1982; King and Horner 1987; Pavón-Vázquez et al. 2022). In 1942, Robert Mertens described a single species (*Varanus acanthurus* BOULENGER) with three subspecies (*V. acanthurus primordius* MERTENS, *V. acanthurus brachyurus* STERNFELD, and *V. acanthurus acanthurus* BOULENGER) (Mertens 1942). Currently, the ridge-tailed goannas are comprised of six species (*V. primordius*, *V. storri*, *V. ocreatus*, *V. insulanicus*, *V. acanthurus*, and *V. citrinus*) (Pavón-Vázquez et al. 2022). They are distinct from all other varanids phenotypically by a ridged tail.

**Fig. 1.**
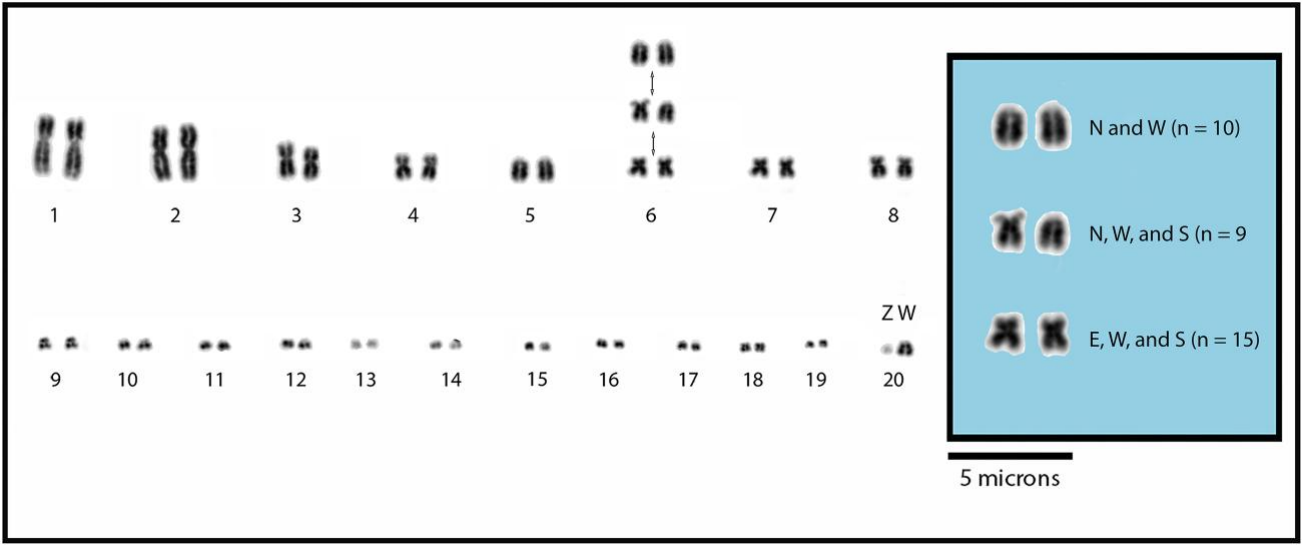
Karyotypes based on staining using standard cytogenetic analysis with DAPI, the chromosome rearrangement occurrence, and differences for chromosome 6 in *V. acanthurus* and *V. citrinus* from four populations. We identified three morphological arrangements for chromosome 6, indicating a polymorphic distribution of AA (top), heterozygous acrocentric submetacentric (middle), and MM (bottom) individuals. Inset shows karyotypes from each locality and the number of individuals with that karyotype. N, northern (*V. citrinus*); W, western (*V. acanthurus*); S, southern (*V. acanthurus*); E, eastern (*V. acanthurus*). The scale bar represents the inset (Figure modified from Dobry et al. 2023a).

The ridge-tailed goannas (*V. acanthurus* complex; Pavón-Vázquez et al. 2022) are a rare example of a wild vertebrate with widespread chromosomal polymorphisms. Chromosomal polymorphisms have been identified in two species, *V. acanthurus* and *V. citrinus* (King et al. 1982; Dobry et al. 2023a). The polymorphisms are described as a pericentric inversion on chromosome 6 and size polymorphisms involving an unpaired enlarged microchromosome and the sex chromosomes (fig. 1) (King et al. 1982; Matsubara et al. 2014; Dobry et al. 2023a, 2023b). Size polymorphisms associated with the sex chromosomes and the enlarged unpaired microchromosome were characterized as a segmental duplication of genes on the W chromosome that likely originated on chromosome 2 (Dobry et al. 2023b). The polymorphisms associated with chromosome 6 exhibit three karyotypes: homozygous submetacentric (MM), heterozygous submetacentric acrocentric (MA), and homozygous acrocentric (AA). The polymorphisms were identified within populations of *V. acanthurus* and *V. citrinus* where gene flow was present within populations and between different karyotypes but not between populations with similar karyotypes (Dobry et al. 2023a). The widespread presence of chromosome polymorphisms and lack of gene flow between populations indicated the inversion spread prior to divergence and reproductive isolation (Dobry et al. 2023a). Further, in the broader outlook for chromosome evolution in Varanidae, the chromosome polymorphism places the ridge-tailed goannas in an intermediate phylogenetic position. The Gouldii clade has fixed acrocentric chromosome 6, and the Varius clade has fixed submetacentric chromosome 6 (King and King 1975). Previous work focused on *V. komodoensis* (Iannucci et al. 2019a, 2019b; Lind et al. 2019), and a comparative study with other varanid species (Iannucci et al. 2019a, 2019b) demonstrated conserved chromosomes across Varanidae. Iannucci et al. (2019a) isolated *V. komodoensis* chromosomes and used chromosome-specific paints across ten different species of varanids. However, chromosomes 6–8 of *V. komodoensis* were flow-sorted into chromosome pools because they were indistinguishable based on size (Pokorná et al. 2016; Lind et al. 2019; Iannucci et al. 2019a). There were two pools, one containing the predicted chromosomes 6/7 and another containing predicted chromosomes 7/8 (Lind et al. 2019; Iannucci et al. 2019a). Consequently, similar-sized chromosomes were pooled together into two pools representing inferred pools for chromosomes 6/7 and 7/8, respectively. The chromosome pools were sequenced and assembled to assign scaffolds for a chromosome-assigned genome assembly (Iannucci et al. 2019a, 2019b). To better understand the implications and role of chromosome polymorphisms and add resolution to the ambiguity of chromosomes 6–8, the major aim of this study was to characterize the polymorphism on chromosome 6 for the two species *V. acanthurus* and *V. citrinus*.

Using an integrated genomics, population genetics, and cytogenetics approach, we showed that multiple chromosome rearrangements occurred independently. These rearrangements have driven DNA sequence divergence along a small portion of the genome involving chromosomes 6 and 7. With a novel approach based on fixed allele analysis, we observed a pattern within or near the centromeric region that distinguishes between chromosome morphologies. Fixed allele differences between populations are a robust way to demonstrate a lack of gene flow (Georges et al. 2018). Therefore, we used this approach to identify the lack of gene flow between chromosome inversion polymorphisms from individuals within a population. We then aligned the population genetics data with two published varanid genomes, *V. komodoensis* and *V. acanthurus*, to identify the location of the single nucleotide polymorphism (SNP) data within the genome (Lind et al. 2019; Iannucci et al. 2019a; Zhu et al. 2022). The alignments allowed for targeting the rearranged chromosome and designing a probe for FISH to test for homology of chromosome polymorphisms between populations. Then we expanded the analysis to other amniotes to demonstrate the synteny of the centromeric region.

## Results

### Karyotype Analysis

All 34 individuals had 2*n* = 40 with 16 macrochromosomes and 24 microchromosomes as described previously (Dutt 1968; King and King 1975; De Smet 1981; King et al. 1982; Srikulnath et al. 2013; Matsubara et al. 2014; Patawang et al. 2017; Iannucci et al. 2019a, 2019b; Augstenová et al. 2021). We observed a macrochromosome polymorphism corresponding to those reported previously (fig. 1) (King et al. 1982; Dobry et al. 2023a). The DAPI-stained karyotypes revealed rearrangements on presumed chromosome 6 in three populations: the northern (*V. citrinus*) and two *V. acanthurus* populations, western and southern. The fourth population (*V. acanthurus*) exhibited a fixed MM morphology east of the Barkly Tableland (table 1 and figs. 1 and 8).

**Table 1.** Summary of Karyotype Differences for 34 Individuals From Different Populations of *V. acanthurus* and *V. citrinus* Around the Barkly Tableland Based on DAPI Staining and Morphological Observations Using Only Cytogenetics.

Karyotype	South (*V. acanthurus*)	West (*V. acanthurus*)	East (*V. acanthurus*)	North (*V. citrinus*)
MM	5	5	5	0
MA	3	3	0	3
AA	0	5	0	5

The chromosome polymorphism was either a pericentric inversion or a centromere repositioning. This type of chromosome rearrangement is consistent with a previous study that used silver staining and characterized the polymorphism as a pericentric inversion (King et al. 1982) (fig. 1).

### Genome Alignments and Identification of Putative Chromosome 6 Scaffolds

**Fig. 2.**
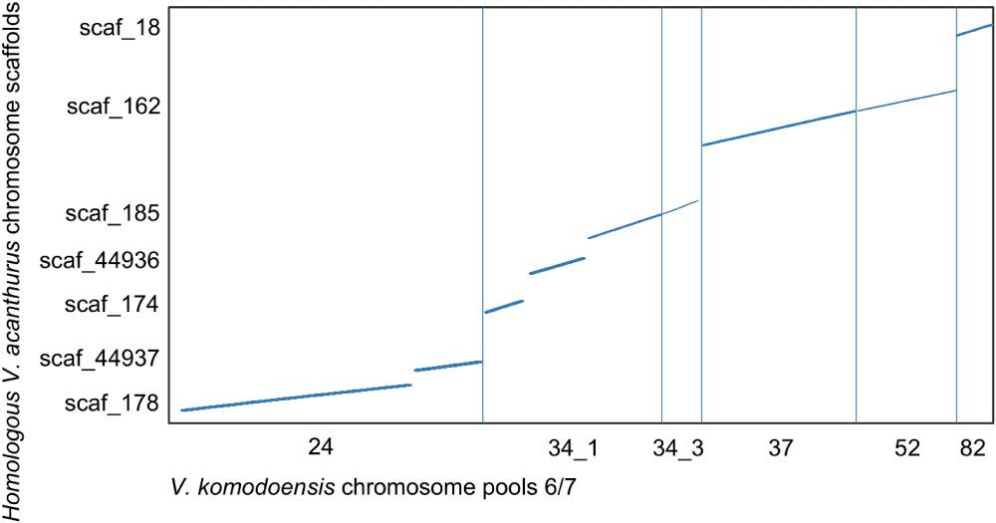
Alignment of *V. acanthurus* scaffolds and *V. komodoensis* scaffolds inferred from flow-sorted chromosome pools representing chromosomes 6 and 7. Labels on the *x*-axis refer to the scaffold IDs from the *V. komodoensis* genome (GCA_004798865.1) and the *y*-axis refers to the scaffold IDs from the *V. acanthurus* genome (BioProjectID PRJNA737594). The horizontal/diagonal lines indicate the synteny blocks between the two genomes.

We identified seven scaffolds from the *V. acanthurus* assembly totaling 83 Mb that aligned to six *V. komodoensis* scaffolds from chromosome pool 6/7 (fig. 2 and supplementary table S1, Supplementary Material online). The *V. acanthurus* scaffolds ranged in size from 2.2 to 46.3 MB, and the homologous *V. komodoensis* scaffolds ranged from 2.6 to 18 MB. The percent identity of the scaffolds ranged from 78 to 100 with a mean identity of 93% and a standard deviation of 2.77.

### Genome Alignments of SNP Data and FISH Probe Design

We analyzed the data set (only those SNPs aligning to putative chromosome 6/7) for fixed allele differences between each karyotype. SNP data from all 34 karyotyped individuals were mapped to the chromosome 6/7 inferred scaffolds of *V. acanthurus*. There were 17,981 out of 301,738 loci mapped to *V. acanthurus* 6/7 scaffolds (table 2). Those 17,981 SNP loci were then mapped to the chromosome 6/7 pools (fig. 2). The fixed allele analysis reduced the SNPs aligning to chromosome 6/7 pools from 17,981 to 45 loci (table 2) where only 45 SNP loci had fixed allele differences.

**Table 2.** SNP Data Alignment With *V. acanthurus* Whole Genome Sequences That Mapped to Chromosome 6/7 Pools of *V. komodoensis* Pre- and Postsorting for Fixed Allele Differences Between Karyotype Morphologies.

*V. acanthurus* Scaffold ID	SNP Loci (presorting)	SNP Loci (postsorting for fixed allele differences)	Scaffold Size in MB
scaf_162	10,089	1	46.3
scaf_174	448	4	2.2
**scaf_178**	**4,057**	**17**	**20.1**
scaf_18	585	0	3.1
scaf_185	1,183	12	5.2
scaf_44936	849	5	3.1
scaf_44937	770	6	3.4

Note.—Scaf_178 (in bold) was chosen as the best candidate for probe development based on the highest fixed allele differences and repeat analysis. Scaf_185 was not included due to the high percentage of CpG islands and low repeat content.

**Fig. 3.**
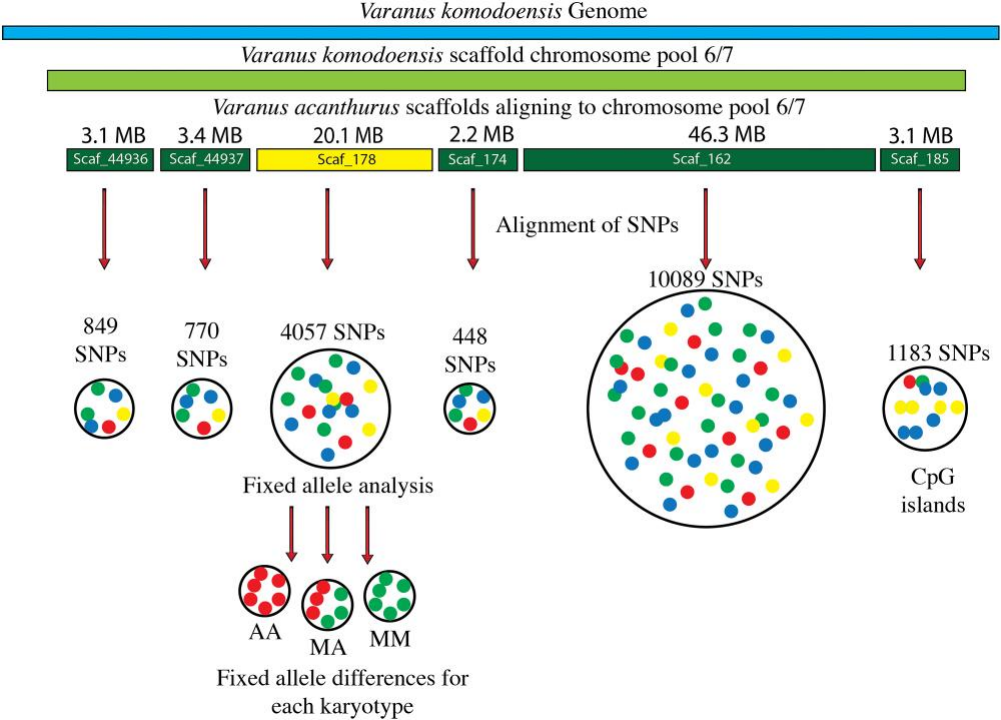
Genome alignments between the *V. komodoensis* and *V. acanthurus* for probe design and alignment of population-specific SNPS for individuals from each karyotype morphology. Fixed allele differences matching karyotype morphologies were mapped to scaf_178, and oligo probes were designed from single-copy regions along this scaffold.

Two scaffolds of interest (scaf_178 and scaf_185) carried the highest count of fixed allele differences between homozygous karyotypes. Evolutionary breakpoint regions are known for increased repeat elements. Therefore, we characterized the repeat elements and used a Wilcoxon test to determine if one of the scaffolds with fixed allele differences also had a significant difference in repeats. The Wilcoxon test demonstrated a significant difference between total repeats, LINE and %GC, but not a significant difference with SINE and simple repeats (supplementary table S2, Supplementary Material online). We designed the chromosome 6-specific FISH probe from scaf_178 because it had the highest total repeats, LINE transposable elements, and the lowest %GC. On the other hand, scaf_185 had the second highest %GC content, was mostly comprised of CpG islands and the lowest total repeats and LINE elements (figs. 3 and S2, Supplementary Material online). The FISH probe consisted of 27,392 individual polynucleotide sequences between 45 and 47 nucleotides in length spanning across 10 Mb of scaf_178 targeting single-copy regions along this 20 Mb scaffold (supplementary table S3, Supplementary Material online).

### Physical Mapping FISH Probes

**Fig. 4.**
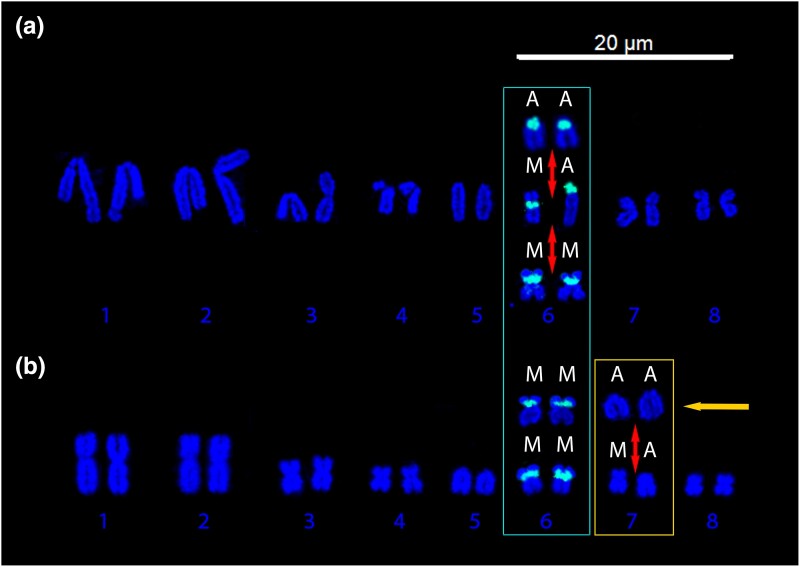
(*a*) Hybridization of the probe to *V. acanthurus* individuals from the south, west, and east populations, which had all three karyotypes, AA, heterokaryotypic (MA), and MM. The red arrows indicate the transition of chromosome morphologies within populations. In these populations, the probe mapped to all morphologies of the polymorphism. (*b*) Hybridization of the probe to chromosome 6 in *V. citrinus* demonstrating that chromosome 6 is fixed submetacentric (MM) in this population and the polymorphisms observed from DAPI staining are a separate chromosome (most likely chromosome 7 indicated by orange arrow) that remains uncharacterized.

The probe was hybridized to 14 individuals (both *V. acanthurus* and *V. citrinus*) containing polymorphisms and fixed arrangements for both homozygous morphologies and heterokaryotypes for chromosome 6, (table 3 and fig. 4) representing individuals from each population.

**Table 3.** Comparison of FISH Results With DAPI Karyotypes for 14 Individuals From Four Populations.

Karyotype	South (*V. acanthurus*)		East (*V. acanthurus*)		West (*V. acanthurus*)		North (*V. citrinus*)	
	DAPI Only	FISH	DAPI Only	FISH	DAPI Only	FISH	DAPI Only	FISH
MM	0	0	2	2	2	2	0	**5**
MA	2	2	0	0	1	1	**3**	0
AA	0	0	0	0	2	2	**2**	0

Note.—Values in bold indicate an inconsistency between DAPI only and FISH results for *V. citrinus*. The probe was homologous for western and southern polymorphisms, but it hybridized to only fixed submetacentric chromosomes in the eastern and northern populations. The northern population, therefore, had novel nonhomologous chromosome polymorphisms that were presumed to be chromosome 6 with DAPI only karyotyping (table 1 and figs. 1 and 8).

The west population had all three morphologies (fixed acrocentric, fixed submetacentric, and heterokaryotypic submetacentric acrocentric), the south had heterokaryomorphic individual heterozygous acrocentric submetacentric, the east had MM, and the north had polymorphisms that were nonhomologous. The probe hybridized to the centromere region of the AA, heterozygous acrocentric submetacentric, and MM chromosomes of all individuals from the west, south, and east as predicted (fig. 4*a*), indicating that we identified the centromere and that it was involved in the rearrangement on chromosome 6. This supported the previous findings of pericentric inversion or centromere relocation for the mode of chromosomal rearrangement (King et al. 1982). However, in *V. citrinus*, the probe only hybridized onto submetacentric chromosomes, revealing that chromosome 6 is fixed submetacentric in this population. This finding contradicts the results from DAPI only karyotyping (figs. 1 and 8 and table 1). Therefore, the heterozygous acrocentric submetacentric and AA chromosomes identified from the *V. citrinus* population are not chromosome 6 and presumed to be chromosome 7 (fig. 4*b*).

### Hypothesizing the Directionality of Chromosomal Change

**Fig. 5.**
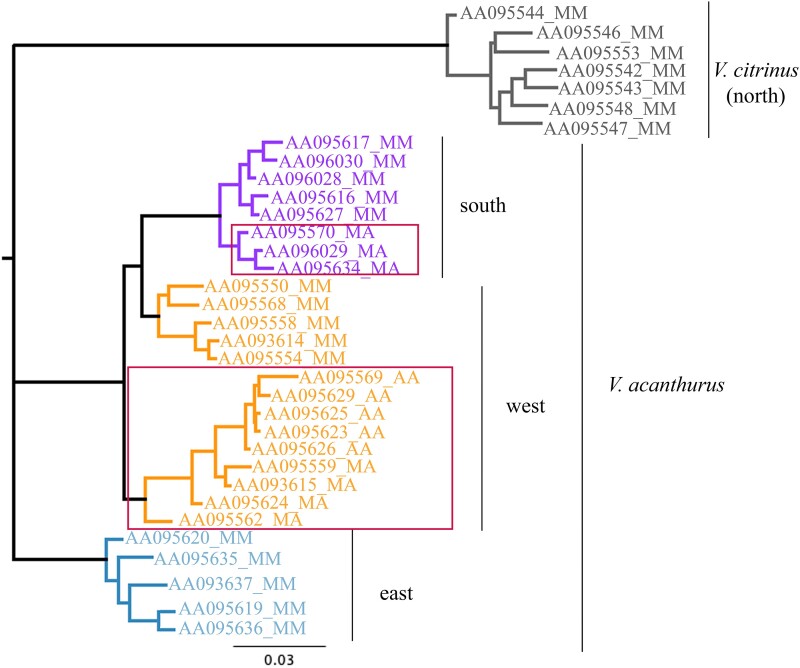
The genetic divergence for centromere scaf_178 from chromosome 6 using PAUP* for maximum likelihood is based on concatenated SNP data generated from DArT and aligned with Geneious Prime 2022.2.1. The tree included 4,057 concatenated SNP markers from all 14 karyotyped individuals for scaf_178. Chromosome polymorphisms (red rectangles) are characterized by MA and AA karyotypes. Chromosome rearrangements are correlated with separate nodes of divergence in two populations, the west (orange) and the south (purple).

After validating that chromosome 6 was fixed submetacentric in *V. citrinus*, we reassigned this karyotype identity to this population and reanalyzed the SNP data set for SNPs aligned to scaf_178. We showed that *V. citrinus* had fixed submetacentric chromosome 6 similar to the east and that the nucleotide divergence based on 4,057 concatenated SNPs suggests submetacentric origin (fig. 5). The submetacentric chromosomes maintain higher genetic diversity along the rearranged scaffold. Due to suppressed recombination in the MA and AA karyotypes, there has been a significant loss of genetic variation in this region (supplementary fig. S3, Supplementary Material online), and the transition from submetacentric to acrocentric has likely occurred independently in both the south and the west, demonstrating that the chromosome rearrangements are points of genetic divergence (fig. 5).

### Reconstructing the History of Chromosome Evolution in Varanidae

**Fig. 6.**
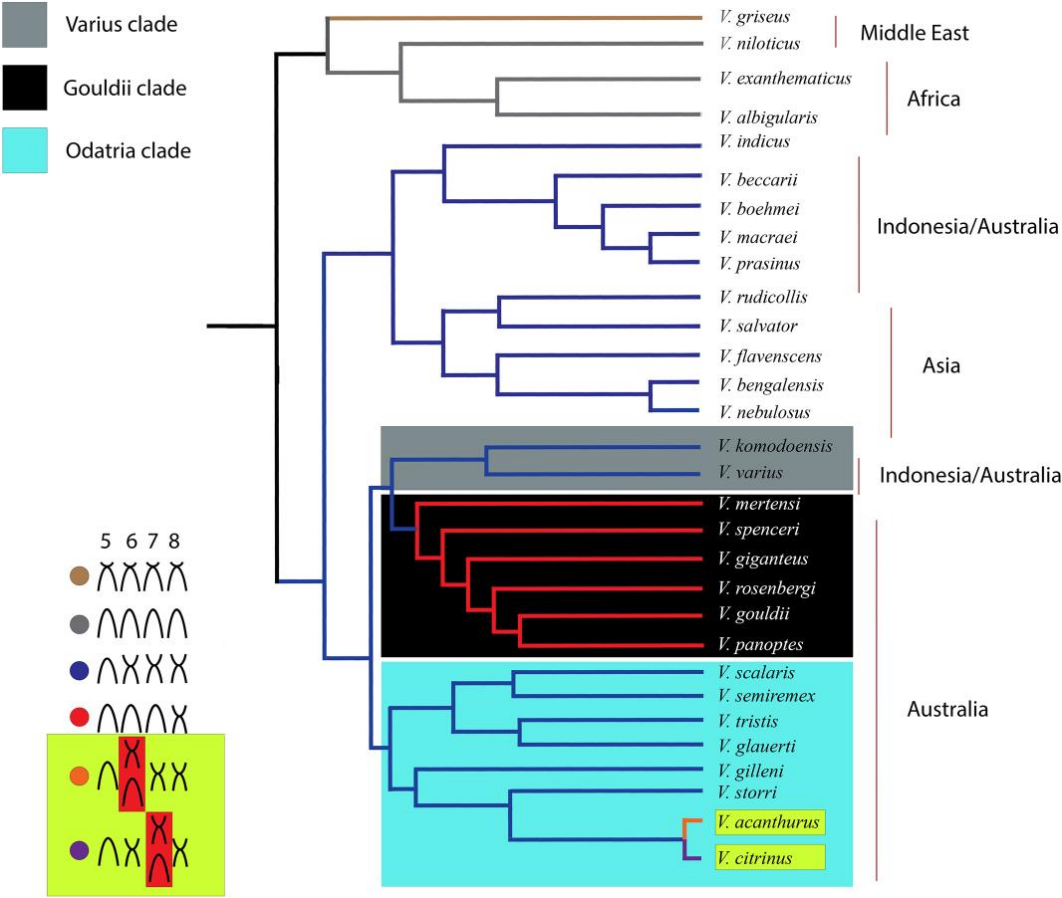
Ancestral reconstruction of the family Varanidae for karyotyped species. The phylogenetic relationships follow Iannucci et al. (2019a, 2019b) for family-wide karyotypes and Brennan et al (2021) based on ASTRAL (Zhang et al. 2018). The node colors reflect each clade's karyotype morphologies for chromosomes 5–8. The three Australian clades are represented with gray (Varius), black (Gouldii), and blue (Odatria) boxes. The karyotypes show the conservation of chromosome morphologies for all clades except Odatria (blue). Within Odatria, *V. acanthurus* and *V. citrinus* are polymorphic for chromosome 6 (*V. acanthurus*) and presumably 7 (*V. citrinus*) and have diverged in the last 1.5 My (Pavón-Vázquez et al. 2022).

We integrated our findings of chromosome rearrangements for *V. acanthurus* and *V. citrinus* with previous reconstructions based on karyotypes (Iannucci et al. 2019a, 2019b) and phylogenies from DNA sequence data (Pyron et al. 2013; Lin and Wiens 2017; Brennan et al. 2021; Pavón-Vázquez et al. 2022). Integration of chromosome transitions with genetic divergence revealed multiple independent chromosomal mutations (fig. 6). This phylogeny showed that a similar transition from submetacentric to acrocentric on both chromosomes 6 and 7 has occurred in the Gouldii lineage, similar to the incipient transition in *V. acanthurus* (chromosome 6) and *V. citrinus* (presumably chromosome 7) within the Odatria lineage (fig. 6).

### Characterizing the Genic Content of Scaf_178

In the published *V. acanthurus* genome, 14,521 protein-coding genes were identified using protein sequences from human and chicken (Zhu et al. 2022). We identified 154 protein-coding genes from scaf_178 which were targeted by the FISH probe from the rearranged region of chromosome 6 (supplementary table S4, Supplementary Material online). OrthoFinder identified 130 genes from *V. acanthurus* scaf_178 for comparison with the other species. A total of 114 orthogroups with all species were present, and 81 of these consisted entirely of single-copy genes. Complete OrthoFinder results are provided as supplementary data, Supplementary Material online (Supplementary Folder OrthoFinder). The genespace visualization showed repositioning of the orthogroups associated with the *V. acanthurus* centromeric region across each species and allowed us to predict the centromere position for each species (table 4). Genespace determines synteny by gene rank order beginning with the rank order of *V. acanthurus* scaf_178. Paralogs are defined as orthologs derived from a duplication event for tandem arrays and are used as anchors (Lovell et al. 2022). Gene rank order is recalculated on these genes masking the copy number and then inferring pairwise these genes as potential anchors with protein BLAST hits where both the query and target genes are in the same orthogroup. Rank orders are calculated prior to the synteny inference and the orthogroups between species are established on a one-to-one relationship for an accurately defined syntenic region (Lovell et al. 2022). Differences in gene rank order allow for identification of inversions and translocations. We compared these putative positions for the putative centromere with published karyotypes for each species. In all cases but two, the general putative centromere position (metacentric vs. acrocentric) matches published karyotypes except for the painted turtle, which could not be determined due to an unassigned scaffold and the western garter snake in which there was no reference karyotype available.

**Table 4.** Basic Cytogenetic Summary for the Taxa Investigated and Predicted Centromere Position Based on FISH Result and Gene Sequence Homology for scaf_178 of *V. acanthurus*.

Species	Common Name	Chromosome Homology with *V. acanthurus* scaf_178	Predicted Centromere Position	Chromosome Morphology	Karyotype Reference	Genome Reference (NCBI accession)
*P. muralis*	Common wall lizard	6	Acrocentric	Acrocentric	Stille et al. 1983; Suwala et al. 2020	GCA_004329235.1
*S. undulatus*	Spiny lizard	4	Metacentric	Metacentric	Cole 1972; Bedoya and Leaché 2021	GCA_019175285.1
*A. carolinensis*	Green anole	4	Metacentric	Metacentric	Gorman 1965; Webster et al. 1972; Giovannotti et al. 2017	GCA_000090745.2
*Z. vivipara*	Common lizard	7	Acrocentric	Acrocentric	Odierna et al. 2001; Kupriyanova et al. 2006	GCA_011800845.1
*T. elegans*	Mountain garter snake	5	Acrocentric	?	No karyotype reference available	GCA_009769535.1
*C. picta*	Painted turtle	8	Unknown due to unassigned scaffold	Metacentric	Badenhorst et al. 2015	GCA_000241765.5
*O. anatinus*	Platypus	4	Acrocentric	Acrocentric	Bick and Jackson 1967; McMillan et al. 2007	GCA_004115215.4
*G. gallus*	Chicken	8	Metacentric or acrocentric	Submetacentric	Krasikova et al. 2006	GCA_016699485.1

The ? under Chromosome morphology for *T. elegans* indicates there was no reference karyotype for this species and the chromosome morphology remains unknown.

## Discussion

The main objective of this study was to molecularly characterize the chromosome polymorphism associated with a transition between acrocentric and submetacentric morphologies of chromosome 6 in the ridge-tailed goannas. Upon identifying the genomic sequence associated with the polymorphisms on chromosome 6, we tested for the homology of the polymorphisms between *V. acanthurus* and *V. citrinus* and revealed that the polymorphisms between these two species were nonhomologous. This study has revealed that those chromosomal polymorphisms within *V. acanthurus* have been derived from fixed submetacentric populations as independent mutations in both the west and south *V. acanthurus* populations. Thus, they are not of similar origin because, in both situations, the most common recent ancestor was submetacentric in origin. Furthermore, we provided putative evidence of the directionality of chromosome change and hypothesized that the chromosome rearrangements are transitioning from submetacentric to acrocentric on chromosome 6 of *V. acanthurus*. This chromosome polymorphism is a dynamic state in at least two recently diverged but isolated populations (Dobry et al. 2023a). A third isolated population maintains the ancestral karyotype (fixed submetacentric). A fourth population, now described as *V. citrinus*, is ancestral according to the most recent genetic phylogeny (Pavón-Vázquez et al. 2022). *Varanus citrinus* maintains the ancestral karyotype for chromosome 6, similar to that of the eastern *V. acanthurus* population but is polymorphic for chromosome 7, a finding that has not been described previously. The characterization of the polymorphism on chromosome 7 in *V. citrinus* remains unresolved. However, our discovery that chromosome polymorphisms are nonhomologous between *V. acanthurus* and *V. citrinus* provides additional evidence that the polymorphisms involving these chromosomes have occurred de novo and are not from a single origin which is a common assumption associated with the spread of chromosome inversions (Wellenreuther and Bernatchez 2018; Faria et al. 2019a, 2019b; Huang and Rieseberg 2020).

### Conserved Chromosomes in Varanidae

Karyotypes in Varanidae are conserved in chromosome number (Deakin and Ezaz 2014; Farré et al. 2016; Damas et al. 2019). This contrasts with other families of lizards, which can vary considerably in the number of chromosomes among species (Deakin and Ezaz 2019). Although varanids are conserved in chromosome number, there is a variation in chromosome morphology (fig. 6). Rearrangements are common in chromosomes 6–8 in Varanidae, and a study that used chromosome paints from *V. komodoensis* showed that all chromosomal variation within Varanidae was intrachromosomal and highly conserved (Iannucci et al. 2019a, 2019b). By cross-species chromosome painting using pools for macrochromosomes, lannucci et al. (2019a, 2019b) compared ten varanid species (two species each endemic to Africa, Asia, and Indonesia and four species from Australia). Their study revealed that all species exhibited conserved sequence homology for chromosome-specific paints across the genome, and there was no evidence of interchromosomal rearrangements. However, chromosomes 6–8 could not be resolved between each other in this study due to their similarity in size (Iannucci et al. 2019a, 2019b). Another study investigated centromeric satellite repeat regions on varanid chromosomes from 17 species (seven Asian, five African, and five Australian [including *V. acanthurus*]). That study showed a lateral movement of repeats between micro- and macrochromosomes, but these patterns were not species specific indicating that these repeats could have independent origins on each chromosome (Prakhongcheep et al. 2017). However, those same repeat subclasses were concordant with centromere placement between fixed chromosome rearrangements on chromosomes 6–8, indicating that these repeats were part of the rearranged regions or played some role in the rearrangement. So far, the ancestral karyotype for Varanidae remains unresolved, and no former studies have been able to demonstrate the directionality of chromosome change in correspondence with phylogenetic reconstructions from DNA sequence data (King and King 1975; Baverstock et al. 1993; Ast 2001; Fitch et al. 2006; Pyron et al. 2013; Srikulnath et al. 2013; Lin and Wiens 2017; Iannucci et al. 2019a, 2019b; Brennan et al. 2021; Pavón-Vázquez et al. 2022).

### Genes Conserved in the Centromere Across Reptilia

**Fig. 7.**
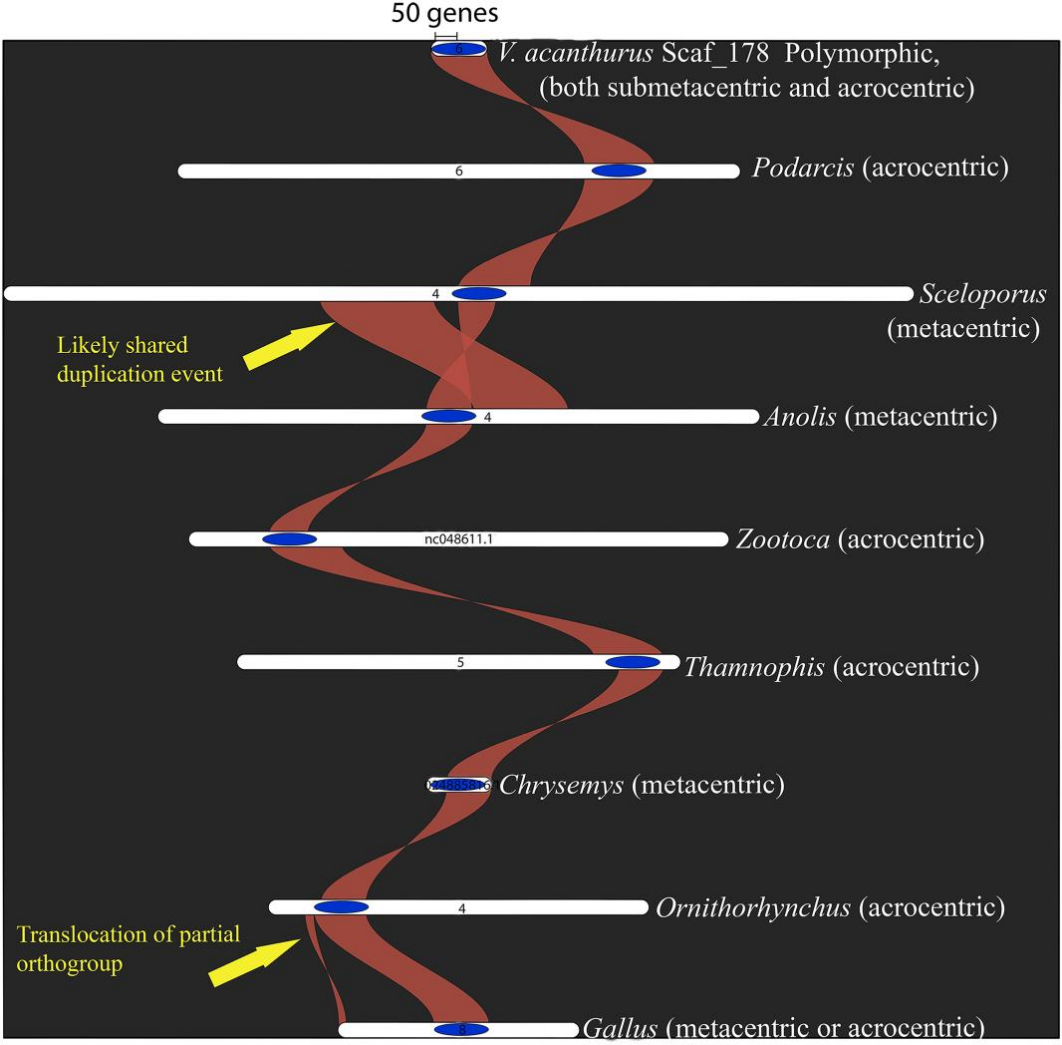
Synteny of the predicted centromere region (blue oval) associated with the relocation of chromosome 6 in *V. acanthurus* with six other reptiles: common wall lizard (*P. muralis*), spiny lizard (*S. undulatus*), green anole (*A. carolinensis*), common lizard (*Z. vivipara*), western terrestrial garter snake (*T. elegans*), painted turtle (*C. picta*), the platypus (*O. anatinus*), and chicken (*G. gallus*). The 20 Mb scaf_178 shows remarkable conservation and appears to be consistently associated with the centromere region in both metacentric or submetacentric chromosomes and acrocentric chromosomes across Reptilia. There appears to be a shared expansion or duplication of the syntenic region between *Anolis* and *Sceloporus* that shows rearrangement, and in chicken, the synteny splits, revealing a partial translocation.

We characterized the synteny of the rearranged section of chromosome 6 and identified the genes involved by comparing them across other Reptilia. The shared orthogroups allowed us to predict the centromere location for all species with reference karyotypes. These findings have implications for the broader understanding of eukaryotic chromosome evolution by identifying the centromere and predicting its placement in all taxa investigated. The complete sequencing of the rearranged centromere and annotated functional genes associated with this region revealed that this rearrangement has the characteristics of an evolutionary breakpoint region and contains genes that are part of homologous syntenic blocks exceeding 250 million years of genetic conservation (fig. 7). This rearranged scaffold is 20 Mb, just over 1% of the 1.4 Gb haploid *V. acanthurus* genome and only 1% of the total genes in a conserved Varanid lineage.

Nevertheless, in this region, we find a mixture of ancient homology and uncharacterized genetic regions representing population-specific and karyotype-specific allele frequencies which mirror genome-wide SNP allele frequencies (Dobry et al. 2023a). Similar patterns between this highly dynamic region of the genome and the rest of the genome indicate that this small region could be a driver of genome-wide divergence or that its allele frequencies are influenced by genome-wide selection. So far, there has been no evidence of lateral gene transfer between chromosomes in the varanid lineage except with satellite repeat regions (Srikulnath et al. 2013; Patawang et al. 2017; Iannucci et al. 2019a, 2019b). How, then, does this small region reflect the divergence pattern of the entire genome? The region is enriched with zinc-finger genes, binding domains, and various genes related to transcription regulation, all characteristics of evolutionary breakpoints (Larkin et al. 2009; Damas et al. 2021). These genes are interlaced with many unique genes and repeat subclasses for which the function remains unknown. We are curious to know if these genes are involved explicitly in local adaptation, various levels of gene expression, or how the chromosome arrangements impact the genes’ fitness or phenotypic expression. We identified specific positions of the scaffold with population-specific SNP loci indicating that divergence along this region is influenced by local selection. Further research focused on functional studies investigating genotypes versus observable phenotypes to determine how genes flanking that region correlate with phenotypes will be necessary to determine how both genomic and chromosomal changes are driven by local selection.

### Genomic Islands Driving Divergence From Within or Near the Centromere

Our results show that de novo divergence patterns have occurred in this 20 Mb scaffold of chromosome 6 that was related to a centromere repositioning (fig. 5). There is mounting evidence that the centromere position is largely under epigenetic control (Altemose et al. 2022). Centromere repositioning could result from CpG methylation (Ichikawa et al. 2017). The elevated genetic substitution rates following CpG loss on the short arm of the submetacentric chromosomes could be the signal we detect as fixed allele differences between the two morphologies. We observed increased fixed allele differences with submetacentric chromosome morphology and CpG islands only on the telomeric region of the long arm (the conserved arm, i.e., not polymorphic) of chromosome 6. However, according to our reference genome (a submetacentric individual), this region also had increased fixed allele differences. Further research is required to support whether hypomethylation is a factor in the nonacrocentric centromere evolution as proposed elsewhere (Ichikawa et al. 2017). To test this, we need to compare the whole genome sequence of an individual with AA chromosomes. Our sequence analysis of the centromeric region showed high levels of unique uncharacterized genetic regions, which can come about in several ways. New genes can evolve from noncoding regions, duplications of existing genes or genomic regions, and coopt from orphaned or retired genes (Rödelsperger et al. 2019). Chromosomal rearrangements are often associated with genome regions with unique and uncharacterized regions (Nieto Feliner et al. 2020). One recent study on ling cod (*Ophiodon elongatus*) showed a divergence between a northern and southern clade originated from a small fraction of the genome, and that portion of the genome was known for rearrangements in the distantly related Atlantic cod (*Gadus morhua*) (Kess et al. 2020; Longo et al. 2020). We observed a similar pattern with substantial divergence along the rearrangement of chromosome 6; this is within or near the centromere subject to epigenetic forces that regulate the stability of this region. Future studies investigating other lineages of varanids with fixed chromosome morphologies and comparing them with the polymorphisms of *V. citrinus* and *V. acanthurus*, which show instability, could illuminate possible mechanisms for large-scale chromosome rearrangements.

Rearrangements are both a source of disease and species divergence. The DNA sequencing-focused efforts of the last couple of decades have revealed surprisingly low predictability between gene sequences and phenotypes, especially with many common diseases and cancers (Ye et al. 2019; Heng and Heng 2022). Many other studies that have identified putative chromosome rearrangements de novo from sequence data rely on patterns of linkage disequilibrium and comparisons between whole genome sequences of individuals with fixed chromosome differences that have been observed cytogenetically from previous work (Fuller et al. 2019; Faria et al. 2019a, 2019b; Huang and Rieseberg 2020; Bedoya and Leaché 2021; Kess et al. 2021). In these studies, the starting data set is genomic, and the rearrangements are often inferred and not validated cytogenetically. Disease research with humans reveals an enigma related to differentiating if patterns of linkage disequilibrium have other causes besides those derived from validated rearrangements. There remains a need for physical mapping to integrate genomic and cytogenetic analysis (Deakin et al. 2019; Liehr 2019, 2021). Often in genomics-only studies, this verification step is overlooked or rarely integrated using the same data set because timelines for genome sequencing and cytogenetics that requires living cells are not easily coordinated with rare samples that are often difficult to obtain. To address this problem, we conducted cytogenetics and genome analysis simultaneously with the same data with the major aim to characterize the rearrangement observed on chromosome 6. Then we mapped the bioinformatically predicted rearrangement to each individual that we had shown cytogenetically had chromosome rearrangements. In doing this, we added resolution to the nature of the chromosome rearrangements revealing that they were nonhomologous and had independent origins.

In conclusion, we achieved our primary aim of characterizing the rearrangement on chromosome 6. We also developed a novel approach for identifying the pericentric region, which revealed its position based on fixed allele differences. We used this method to identify and characterize the de novo rearrangements within populations and other reptiles. Our analysis also revealed that a sister species *V. citrinus* has a second chromosome polymorphism unrelated to chromosome 6, demonstrating that chromosome rearrangements in this group are more complex than previous reports (King et al. 1982; Dobry et al. 2023a). We have shown that some populations of *V. acanthurus* have maintained ancestral chromosome morphologies for one pair of chromosomes and derived for another leading to islands of divergence that represent only 1% of the genome. Despite this observation of extreme divergence within this region, it maintained highly conserved genes and was consistently associated with the centromere region in all taxa investigated, from turtles to chickens. These findings have implications for the broader understanding of eukaryotic evolution and investigating relationships with chromosome rearrangements and phenotypes associated with disease. Our research also demonstrates the importance of generating high-quality physical maps integrated with cytogenetic and genomic data sets to resolve genome complexity that cannot be addressed with DNA sequencing or cytogenetic analysis alone.

## Materials and Methods

### Ethical Approval

This research was conducted under the approval of the Animal Ethics Committee at the University of Canberra as mandated in the ACT Animal Welfare Act 1992. The project identification number was 20180306. Separate permits were required for the collection of animals. They were issued by Northern Territory Parks and Wildlife Commission, permit number 63414, and Queensland Government Department of Environment and Science, permit number WA0010049. The animals were imported to the ACT and maintained under the license number LT201829.

### Collection of Specimens

**Fig. 8.**
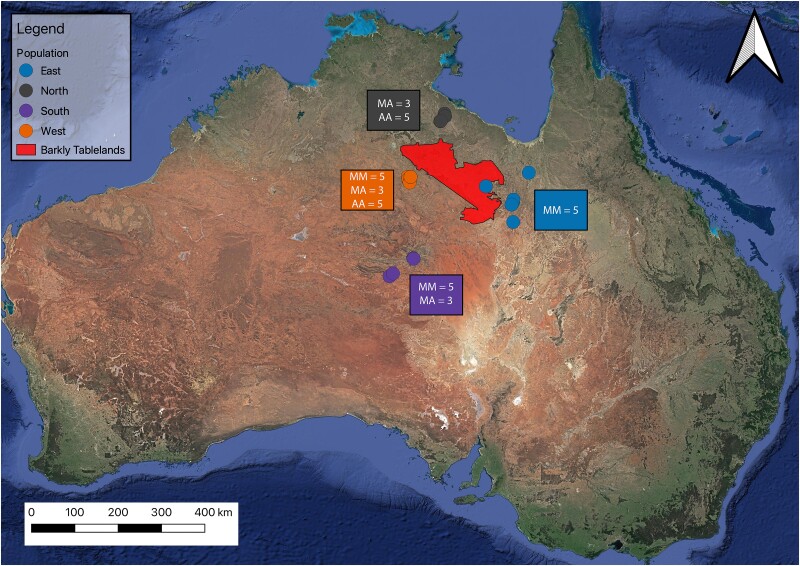
Karyotype variation within *V. acanthurus* collections based on cytogenetic analysis. Homozygous acrocentric chromosomes are represented by AA, MA represents heterozygous submetacentric acrocentric, and homozygous submetacentric chromosomes are MM. The map is modified from Dobry et al. (2023a).

The geographic distinction between fixed submetacentric and polymorphic populations was along the Barkly Tableland (King et al. 1982). Therefore, collection efforts targeted populations in this region. Individuals were collected by hand when sheltering among rocks deposited by road graders along roadsides in the Northern Territory and Queensland (fig. 8). Upon collection, the animals were placed into muslin bags, into an insulated box, and freighted overnight to the Canberra Airport. The individuals were then transported to the University of Canberra and housed in terrariums as described by Retes and Bennett (2001). A laboratory colony was established with these individuals from four distinct geographical populations with known chromosome rearrangements (King et al. 1982). From each individual, we collected blood for DNA extraction and tail tissue for chromosome preparation. Individuals were morphologically identified to species and then genetically profiled using SNP markers and compared with museum samples from a separate study that included several species in the ridge-tailed goanna species complex (Pavón-Vázquez et al. 2022).

### Cell Culture, Chromosome Preparations, and Karyotype Analysis

Cell cultures were established from 34 individuals, as described in Dobry et al. (2023a). The animals were washed with chlorhexidine soap, and any old scales were removed. A sterile scalpel was used to remove ∼1 cm of the tail tip. The tail tissue was then soaked in 6% (*v*/*v*) hydrogen peroxide solution for 5 min and then washed with Betadine before macerating in Hanks Balanced Salt Solution (Sigma-Aldrich Corp., Milwaukee, WI, USA) with 1× antibiotic–antimycotic (Thermo Fisher Scientific Australia Pty Ltd., Scoresby, Victoria, Australia). Metaphase chromosomes were prepared with standard methods as described elsewhere (Ezaz et al. 2005, 2008; Matsubara et al. 2014). Slides were prepared with 10 µL of cell suspension dropped onto each slide from a height of 20 cm, allowed to dry, washed with 100% ethanol, and stained using Vectashield antifade mounting medium with DAPI (Vector Laboratories). Slides were viewed and photographed with a Zeiss Axio Scope A1 epifluorescence microscope equipped with an AxioCam MRm Rev. 3 (Carl Zeiss Ltd., Cambridge, UK) camera and Metasystems Isis FISH Imaging System V 5.5.10 (Metasystems, Newton, MA, USA) software.

### Genome Alignments, Population Genetics Analysis, and Probe Design

We aligned two publicly available varanid genomes, *V. komodoensis* (NCBI assembly GCA_004798865.1; Lind et al. 2019) and *V. acanthurus* (raw sequences deposited with NCBI BioProjectID PRJNA737594 and full assembly available with Genome Warehouse under accession PRJCA005583; Zhu et al. 2022). The *V. acanthurus* genome was aligned with flow-sorted chromosome pool contigs/scaffolds for chromosomes 6/7 from *V. komodoensis* (Lind et al. 2019; Iannucci et al. 2019a) using Nucmer (v4.0.0beta2) with parameters -b 500 (fig. 2). Only one-to-one synteny blocks with lengths >500 bp were retained for analysis. We focused on the 6/7 chromosome pools to reduce the complexity of the genome and target the predicted chromosome rearrangement, which was hypothesized to be chromosome 6 in all populations (King et al. 1982; Dobry et al. 2023a). All individuals were sequenced by Diversity Arrays Technology (DArT, Bruce, ACT, Australia) for SNP analysis (Dobry et al. 2023a). DArT is a genome complexity reduction technology that utilizes restriction enzymes to fractionate the genome and incorporates Illumina sequencing to characterize the SNPs associated with the genome fractions (Kilian et al. 2012). We aligned the Illumina reads containing SNPs against the *V. acanthurus* scaffolds using BLAST+ version 2.13. For the BLASTn analysis, we used minimum 3% and maximum 60% for each base. Then BLASTn *e* value was 5e^−7^ with culling limit of 200, maximum hits per sequence was 1, with a 70% sequence overlap, and 70% sequence identity. We used the dartR package version 2.7.2 for further downstream analysis of the SNP data and chromosome scaffold alignments (Gruber et al. 2018; Mijangos et al. 2022). We sorted the SNP data to analyze the data aligning only to the putative chromosome 6/7 scaffolds. To avoid population-specific differences confounding our analysis for chromosome homology between populations, we used the western population as a reference because it was the only population with all three karyotype morphologies, and gene flow was present despite karyotype differences (Dobry et al. 2023a).

**Fig. 9.**
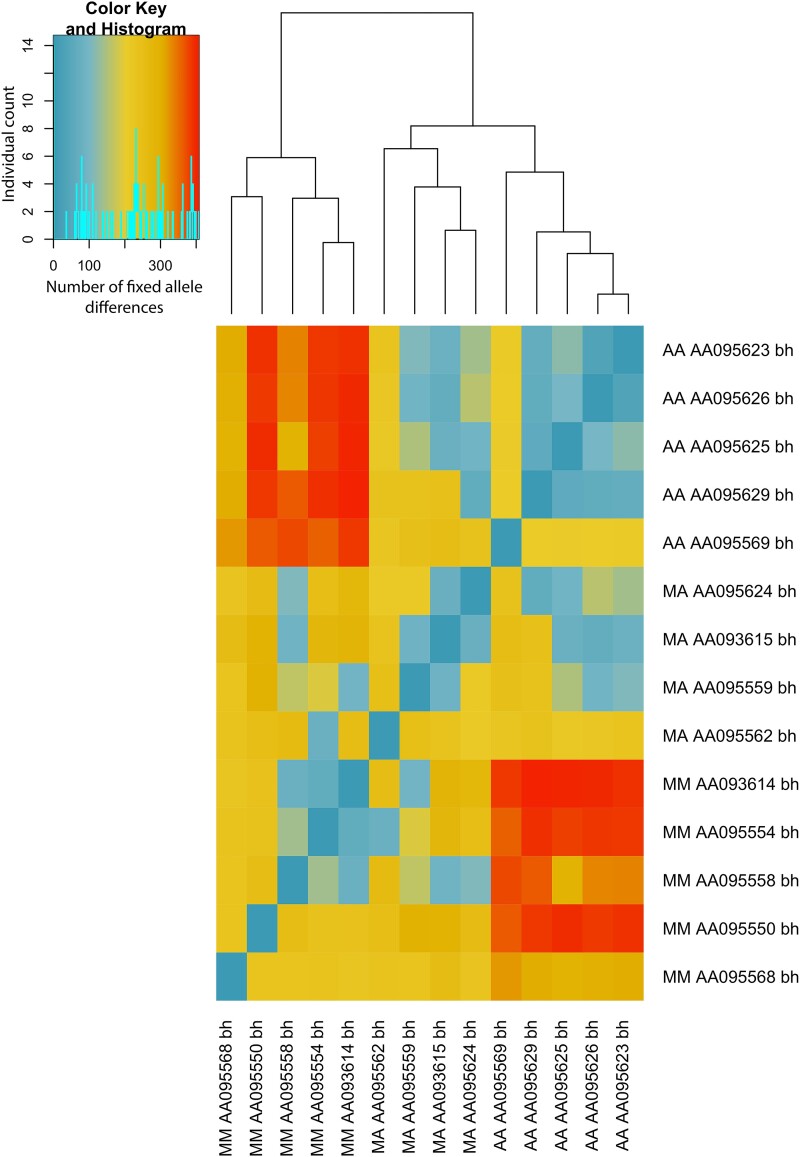
Heat map of fixed allele differences between individuals with polymorphic karyotypes. Individuals are represented by their karyotype, MM, AA, and heterokaryotypes (MA), followed by the specimen ID (AA number) and the population ID (bh). The Barkly Highway population in the west (bh) was the only population with all three karyotypes. To test for recombination suppression between chromosomes, we used fixed allele differences, a robust measure of lack of gene flow. The heat map depicts that increased fixed allele differences (red squares) were associated with the MM karyotypes, and the least fixed allele differences (blue squares) were found on the AA karyotypes. The MA individuals (heterokaryotypes) were intermediates (mostly yellow and blue squares) between the AA and MM individuals. The values of heterokaryotypes indicated that shared alleles were found between the AA and heterokaryotypic individuals. However, when an individual had two submetacentric chromosomes, there was a significant increase in the fixed allele differences. The dendrogram at the top of the figure used hierarchical clustering to demonstrate Euclidean distances of fixed alleles between individuals.

We used fixed allele analysis of individuals for each chromosome morphology to identify the specific rearranged region among the scaffolds comprising the chromosome 6/7 alignments. We observed a pattern of fixed differences between homozygous metacentric chromosomes and AA chromosomes (fig. 9). The DArT data set denotes the SNP locus by calling alleles to 0, 1, and 2, where 0 is a homozygous state, 1 is heterozygous, and 2 is homozygous for the opposite allele. Therefore, to identify the loci with fixed allele differences between karyotypes, we developed an R script that associated 0 or 2 with AA karyotypes and the opposite calls 2 and 0, respectively, for the MM karyotypes. Heterokaryotypes had a 1 for the corresponding loci. The R script generated a table of the Illumina sequences containing the individual SNPs and the corresponding *V. acanthurus* genome scaffolds aligned with the reads (tables 2 and S5, Supplementary Material online).

To characterize the scaffolds for repeat content, first, we used RepeatMasker (v4.0.7) (Tarailo-Graovac and Chen 2009) with parameters -xsmall, -species, squamata – pa 40 -e ncbi, and then Repbase (v21.01) to annotate the repeat sequences (supplementary table S6, Supplementary Material online). We then illustrated the repeat content with Circos version 0.69-8 (Krzywinski et al. 2009) for the scaffolds aligning to the chromosome 6/7 pools of *V. komodoensis*. All repeat densities were reduced to 100 K windows, with the maximum proportion normalized to 1. In addition to the visualization of the repeats, we used a Wilcoxon test (Bauer 1972) to compare the different repeat elements between scaffolds 178 and 185 (hereafter scaf_178 and scaf_185) and determine if there was a significant difference (supplementary table S2, Supplementary Material online).

To determine the gene content for scaf_178, we used the protein libraries for human (GCA_000001405.28), chicken (GCA_016699485.1), duck (GCA_002743455.1), and zebra finch (GCA_003957565.2). The threshold used was identity >30 and *e* < 1e^−10^. Genes that did not give BLAST results or were retired transcripts were considered uncharacterized genes (supplementary table S4, Supplementary Material online).

We purchased the probe set from Arbor Biosciences (myTags, Arbor Biosciences, Ann Arbor, MI, USA) which was designed from scaf_178. The probe set was a population of 27,387 biotinylated oligonucleotides that spanned 10 MB of scaf_178 of the *V. acanthurus* genome (supplementary fig. S1 and table S3, Supplementary Material online). This section of scaf_178 was the mapped location of the fixed allele differences between AA and MM chromosomes. These probes were specific to single-copy regions along scaf_178. The *V. komodoensis* genome assembly was used as a surrogate for the *V. acanthurus* genome to determine probe specificity at the genomic scale. First, the scaffold was BLASTed against the *V. komodoensis* genome assembly (NCBI accession GCA_004798865.1; Lind et al. 2019) to identify homologous regions (word size of 21 and *e* value of 1e^−50^). Those regions were excluded from the *V. komodoensis* genome assembly to prevent confusion with the input *V. acanthurus* sequences during probe design. A hybrid genome assembly was generated by concatenating the modified *V. komodoensis* reference genome and the input scaffolds from the *V. acanthurus* genome (NCBI BioProjectID PRJNA737594, Genome Warehouse BioProject accession PRJCA005583; Zhu et al. 2022). The *V. acanthurus* input scaffolds of interest were cut into overlapping probe candidates tiled every three nucleotides. Probe length varied from 43 to 45 nucleotides to minimize their thermodynamic property range. Probe candidates were checked against the hybrid genome assembly using Arbor Biosciences’ proprietary thermodynamic-based software for their ability to hybridize only to their specific targets without hybridizing to other regions of the genome under usual hybridization conditions.

### FISH of Custom Oligo Probes

To hybridize the probe, we followed the manufacturer's recommendations. In brief, we resuspended the probe to a concentration of 100 ng/µL. We used 200 ng per slide diluted into 38 µL hybridization buffer (BioCare Medical, Pacheco, CA), and then coverslips were sealed with rubber cement. The slides were denatured at 68 °C for 5 min and then incubated at 37 °C for 24–48 h. Following incubation, the coverslips were removed, and the slides were washed with 0.4× SSC: 3 M NaCl, 0.3 M sodium citrate, pH 7, and 0.3% (*v*/*v*) IGEPAL (Sigma-Aldrich) at 60 °C for 2 min, followed by a second wash at room temperature with 2× SSC: 3 M NaCl, 0.3% M sodium citrate, pH 7, and 0.1% (*v*/*v*) IGEPAL (Sigma-Aldrich) for 1 min. The slides were then desiccated with an ethanol wash series of 70%, 90%, and 100% (*v*/*v*) for 1 min each and allowed to dry completely. Once dry, the slides were stained with Vectashield antifade mounting medium with DAPI (Vector Laboratories) and viewed and photographed with a Leica Microsystems Thunder Imaging system. Karyotype images were constructed from metaphase chromosomes using Adobe Photoshop 2021.

### Reconstruction of the Ancestral Karyotype and Direction of Chromosome Change

We concatenated the SNPs from the DArT Illumina reads, which aligned to scaf_178 (table 2) using the dartR package version 2.7.2 (Gruber et al. 2018; Mijangos et al. 2022). The concatenated SNPs generated a nucleotide sequence for each individual that had been karyotyped. The allele tags from DArT are unphased; therefore, we replaced heterozygous positions with standard ambiguity codes and used the concatenated SNPs to generate a single sequence across all loci for that scaffold generating a single sequence for each individual. We then aligned these sequences with Muscle 3.8.425 (Edgar 2004) and used PAUP* 4.0a and Geneious Prime 2022.2.1 (Wilgenbusch and Swofford 2003) to generate a maximum likelihood tree for SNP loci with default parameters and a midpoint root method (fig. 5). To demonstrate the lack of genetic diversity on AA chromosomes, we filtered the SNP loci for a call rate of 1 to remove all missing loci between populations and generated a smear plot (supplementary fig. S3, Supplementary Material online).

### Characterizing Scaf_178 and Determination of Synteny With Other Reptiles

We subset the protein coding genes identified from scaf_178 and used OrthoFinder version 2.5.4 (Emms and Kelly 2015, 2019) and genespace version 0.9.3 (Lovell et al. 2022) with default parameters for synteny visualization across species. These protein sequences and the corresponding DNA sequences were used as input for OrthoFinder (Emms and Kelly 2015, 2019, 2020) and then genespace (Lovell et al. 2022) for visualization with pairwise comparisons against six other chromosome-level genome assemblies from other reptiles. We included the following species: common wall lizard (*Podarcis muralis*, NCBI accession GCA_004329235.1), spiny lizard (*Sceloporus undulatus*, NCBI accession GCA_019175285.1), green anole (*Anolis carolinensis*, NCBI accession GCA_000090745.2), common lizard (*Zootoca vivipara*, NCBI accession GCA_011800845.1), western terrestrial garter snake (*Thamnophis elegans*, NCBI accession GCA_009769535.1), painted turtle (*Chrysemys picta*, NCBI accession GCA_000241765.5), the platypus (*Ornithorhynchus anatinus*, NCBI accession GCA_004115215.4), and chicken (*Gallus gallus*, NCBI accession GCA_016699485.1) (fig. 7).

## Supplementary Material

Supplementary data are available at *Molecular Biology and Evolution* online.

## Supplementary Material

msad124_Supplementary_Data

## Data Availability

The raw DArT Illumina files are uploaded to NCBI under accession PRJNA949379. The .csv files and relevant rcode for the DArT analysis are uploaded to Dryad. The Dryad data set also includes the repeat element files for the Circos image and the Wilcoxon test, the peptide and fasta files for the genespace, and the .rmd file used for this analysis; the full data set for the OrthoFinder and genespace runs are included in .zip files; and the *V. acanthurus* scaffolds for chromosome 6_7 are provided. The study’s data set can be accessed in Dryad under https://doi.org/10.5061/dryad.6djh9w14z.
