# Supplementary material for: Fixed Allele Differences Associated With the Centromere Reveal Chromosome Morphology and Rearrangements in a Reptile (Varanus acanthurus BOULENGER)
Source: Mol Biol Evol. 2023 Jun 5;40(6):msad124. doi: 10.1093/molbev/msad124 (PMC10257493; doi:10.1093/molbev/msad124)
Supplement: msad124_Supplementary_Data [file msad124_supplementary_data.zip › Supplementary Figures.pdf]

Supplementary Figures

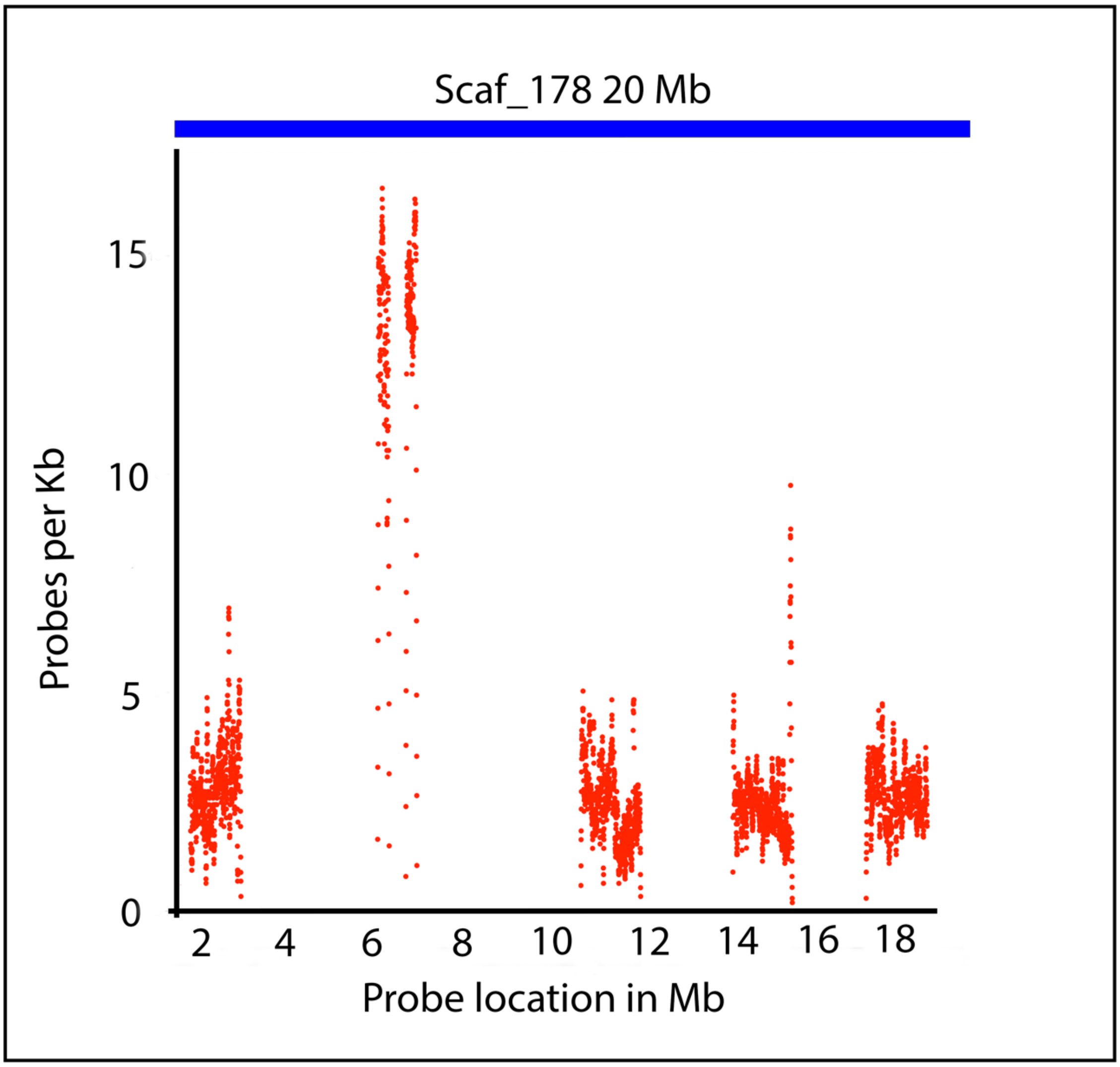

Supplementary Figure 1. Oligo probe distribution along scaf_178. The X-axis displays the probe location in Megabases along scaf_178. The Y-axis is the probe density in number of probes per Kilobase. Regions along scaf_178 with no probe are high in repeats or have homology with other parts of the genome.

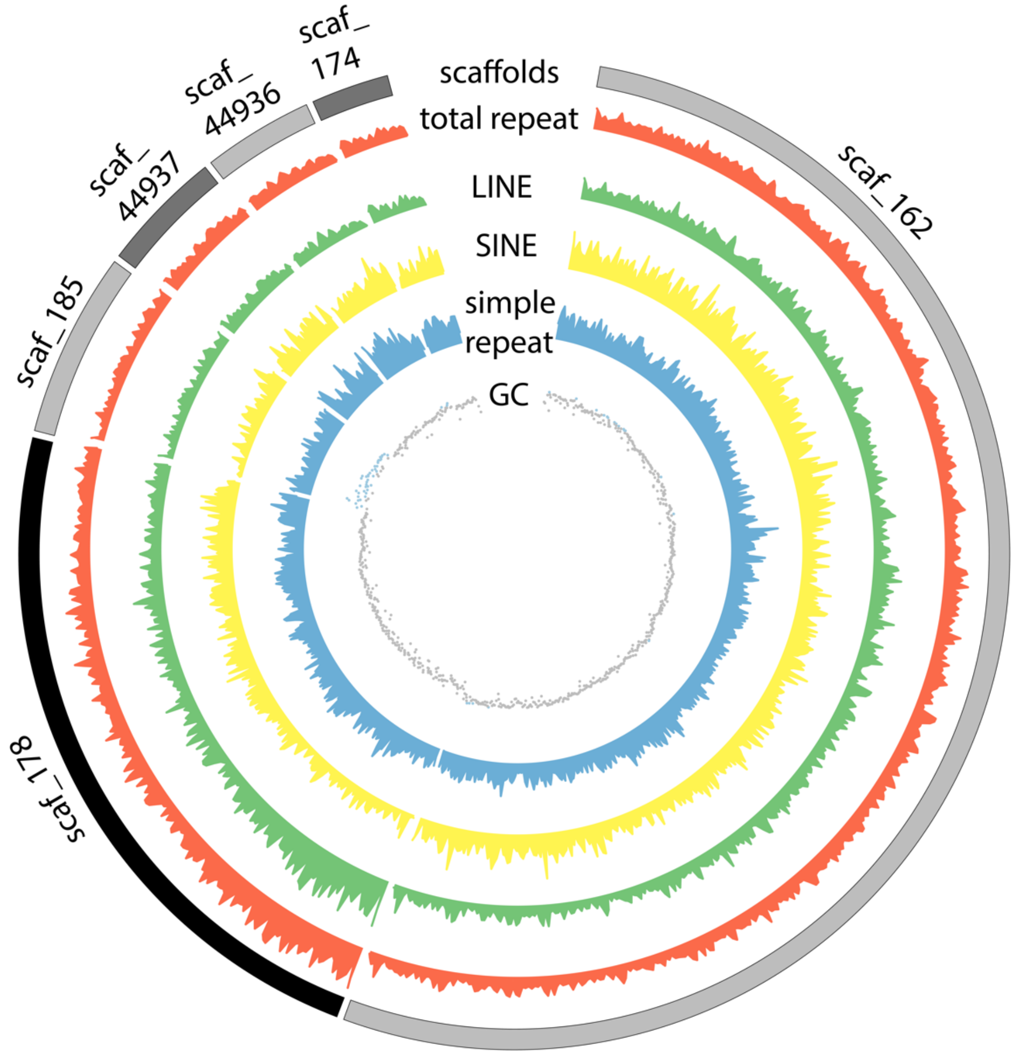

Supplementary Figure 2. Circos v0.69-8 image of repeat regions for the scaffolds from the chromosome 6/7 pools. Scaffolds are indicated with grey/black. Total repeats are red, LINE elements are green, SINE elements are yellow, simple repeats are blue, and % GC are grey.

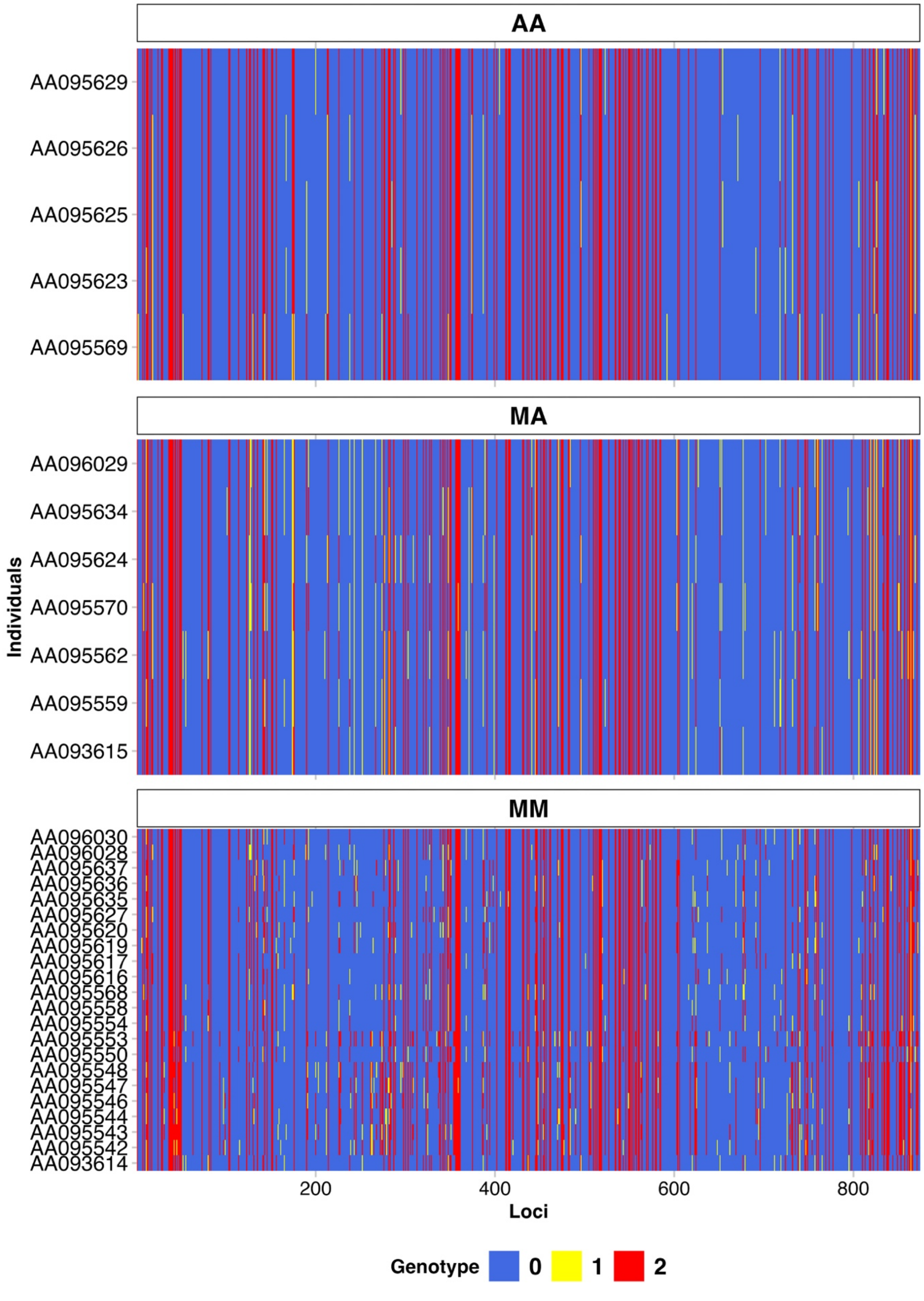

Supplementary Figure 3. Smearplot of scaf_178 for each karyotype for 866 SNP loci. Scaf_178 was filtered for a call rate of 1 to eliminate any NA loci that were population specific. SNP loci are indicated on the X-axis. Individuals are indicated by the AA numbers on the Y-axis for each plot. AA is homozygous acrocentric, MA is heterokaryotypic

submetacentric acrocentric, and MM is homozygous submetacentric. The genotypes are blue for 0 (homozygous for 1 allele), yellow for 1 (heterozygous), and red for 2 (homozygous for the opposite allele). Submetacentric karyotypes (MM) carried higher genetic diversity compared to heterokaryotypic and homozygous acrocentric karyotypes.
